# Development of a Dry-Reagent-Based qPCR to Facilitate the Diagnosis of *Mycobacterium ulcerans* Infection in Endemic Countries

**DOI:** 10.1371/journal.pntd.0003606

**Published:** 2015-04-01

**Authors:** Jérémie Babonneau, Christian Bernard, Estelle Marion, Annick Chauty, Marie Kempf, Raymond Robert, Laurent Marsollier, Franco-Beninese Buruli Research Group Benin:

**Affiliations:** 1 ATOMycA, Inserm Avenir Team, CRCNA, Inserm U892, 6299 CNRS and LUNAM, CHU and Université d'Angers, Angers, France; 2 SR^2^B, Avrillé, France; 3 Centre de diagnostic et de traitement de l'ulcère de Buruli, Pobè, Bénin; 4 Laboratoire de Bactériologie, Institut de Biologie en Santé—PBH, CHU d’Angers, Angers, France; University of Tennessee Knoxville, UNITED STATES

## Abstract

**Background:**

Buruli ulcer is a neglected tropical disease caused by *Mycobacterium ulcerans*. This skin disease is the third most common mycobacterial disease and its rapid diagnosis and treatment are necessary. Polymerase chain reaction (PCR) is considered to be the most sensitive method for the laboratory confirmation of Buruli ulcer. However, PCR remains expensive and involves reagents unsuitable for use in tropical countries with poor storage conditions, hindering the development of reliable quantitative PCR (qPCR) diagnosis. We aimed to overcome this problem by developing a ready-to-use dry qPCR mix for the diagnosis of *M*. *ulcerans* infection.

**Methodology/Principal Findings:**

We compared the efficiency of three different dry qPCR mixes, lyophilized with various concentrations of cryoprotectants, with that of a freshly prepared mixture, for the detection of a standard range of *M*. *ulcerans* DNA concentrations. We evaluated the heat resistance of the dry mixes, comparing them with the fresh mix after heating. We also evaluated one of the dry mixes in field conditions, by analyzing 93 specimens from patients with suspected Buruli ulcers. The dry mix was (i) highly resistant to heat; (ii) of similar sensitivity and efficiency to the fresh mix and (iii) easier to use than the fresh mix.

**Conclusions:**

Dry qPCR mixes are suitable for use in the diagnosis of *M*. *ulcerans* infection in endemic countries. The user-friendly format of this mix makes it possible for untrained staff to perform diagnostic tests with a limited risk of contamination. The possibility of using this mix in either vial or strip form provides considerable flexibility for the management of small or large amounts of sample. Thus, dry-mix qPCR could be used as a reliable tool for the diagnosis of Buruli ulcer in the field.

## Author Summary

Buruli ulcer is a neglected tropical disease caused by *Mycobacterium ulcerans* that may result in severe skin ulceration if left untreated. Quantitative PCR (qPCR) is the most sensitive method for diagnosing Buruli ulcer, but is difficult to perform in endemic areas. We overcame these problems by developing an easy-to-use, heat-resistant dry-reagent qPCR device.

## Introduction

Buruli ulcer, caused by *Mycobacterium ulcerans*, is a necrotizing skin disease mostly affecting children under the age of 15 years, and often leading to permanent disabilities[1]. It is one of the 17 neglected tropical diseases identified by the WHO and is the third most common mycobacterial disease affecting humans, after tuberculosis and leprosy [2,3]. Buruli ulcer is endemic to swampy rural areas of tropical and subtropical countries, principally in Africa, although it has been reported in more than 30 countries [2,3].

The early stages of the disease are characterized by a painless nodule, papule, plaque or edema. If left untreated, these lesions progress to the characteristic painless ulcer with undermined edges. Small lesions are treated with a combination of rifampicin and streptomycin for eight weeks [1,4,5]. Surgery may be required in addition to this antibiotic treatment, particularly if the lesions are extensive.

The diagnosis of Buruli ulcer can be confirmed by several methods: (i) direct smear examination, to detect acid-fast bacilli, is a rapid test that is simple to perform but of low sensitivity; (ii) culture on Lowenstein Jensen medium at 32°C; this is the most discriminatory method, but is not very sensitive and takes more than eight weeks, rendering it of little use to clinicians; (iii) histopathological examination is sensitive but expensive and requires a sophisticated laboratory, well trained personnel and invasive procedures (biopsy); (iv) polymerase chain reaction (PCR)-based IS*2404* detection is the most widely used method. It is highly sensitive and specific and is also reasonably rapid, but it is expensive and requires trained personnel with specific equipment [6,7,8].

The WHO recommends that PCR confirmation should be performed for at least 70% of the cases reported for any district or country [9,10]. African molecular biology laboratories have recently been equipped with real-time thermal cyclers for the management of many diseases, including avian influenza. This material could be used to diagnose many neglected tropical diseases, including Buruli ulcer. However, due to the lack of a ready-to-use PCR tool, molecular diagnosis is often delayed, because the samples must be sent to reference laboratories, which are frequently located far from Buruli ulcer treatment centers. There may also be significant difficulty obtaining and storing expensive reagents.

In this context, as already done for conventional PCR [11,12], we developed a ready-to-use dry mix, containing all the reagents (master mix, primer and probes) required for quantitative PCR (qPCR) and a mixture of collagen and trehalose as cryoprotectants (dry qPCR mix), for the diagnosis of *M*. *ulcerans* infection, suitable for use in the countries in which this bacterium is endemic. The resistance of this dry mix to heat and its stability for long-term storage overcome the shipping and storage constraints, and the ready-to-use format simplifies the handling of the test and facilitates qPCR diagnosis, potentially leading to direct improvements in the medical care of Buruli ulcer patients.

## Materials and Methods

### Ethics statement

Ethical approval for the analysis of specimens from patients was obtained from the institutional review boards of the CDTLUB of Pobè and Angers University Hospital. The specimens used for this study were anonymized.

### DNA extraction and purification

Tissue specimens were finely chopped and homogenized in sterile water with a Tissue Lyser II (Qiagen). Swabs were homogenized in sterile water, and fine-needle aspirates (FNAs) were used directly. For DNA extraction, 400 μl of sample was centrifuged at 3000 x *g* for 10 minutes. The supernatant was discarded and the pellet was washed twice with 800 μl of DNAse-free water per wash, with centrifugation at 3000 x *g* for 10 minutes to recuperate the pellet.

The supernatant was discarded and the pellet was suspended in 50 μl of 50 mM NaOH and heated at 95°C for 20 minutes to lyse the bacteria. DNA was purified with a QIAquick purification kit (Qiagen), after adjusting the pH by adding 10 μl of 3 M sodium acetate pH 5.2, according to the manufacturer’s recommendations.

### PCR conditions

#### Fresh qPCR mix composition

Oligonucleotide primers and *Taq*Man probes were selected based on the IS*2404* sequence (S1 Table) [7,13]. The qPCR mixture contained 0.3 μM of each primer, 0.1 μM probe, a glycerol-free Master Mix (SuperHotTaq Master Mix 2X, BIORON) containing 5 mM MgCl_2_ and 5 μl of template DNA, in a total volume of 25 μl. Amplification and detection were performed with a thermal cycler (Chromo4, Bio-Rad) using the following program: heating at 95°C for 10 minutes, followed by 40 cycles of 95°C for 15 s and 60°C for 1 minute. The results were analyzed with Opticon Monitor software (Bio-Rad).

#### Dry qPCR mix preparation

**Fig 1:**
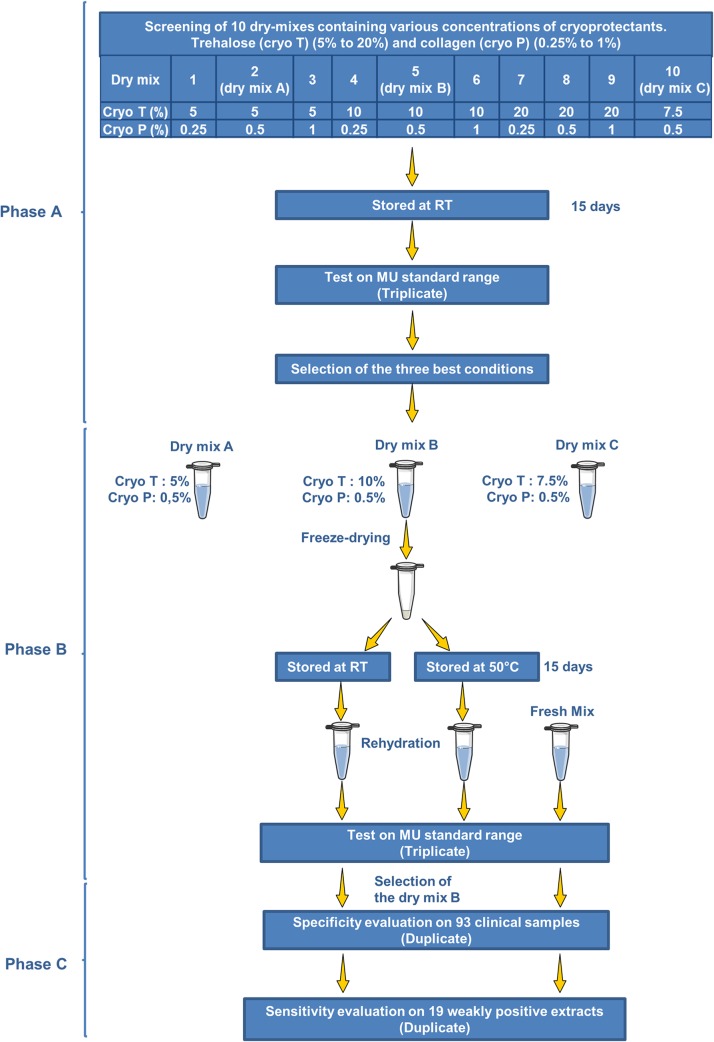
Diagram of the study.

Various dry PCR mixtures were prepared and evaluated for efficiency and accuracy. The dry mixes contained 0.3 μM of each primer, 0.1 μM probe, and a glycerol-free Master Mix (SuperHotTaq Master Mix 2X, BIORON) containing 5 mM MgCl_2_. They were freeze-dried as described in patent no. EP1629118 (A2), using two cryoprotectants, collagen (cryo P) and trehalose (cryo T). The minimum and maximum concentrations of the cryoprotectants compatible with the chosen qPCR technique were determined during a preliminary evaluation. We used concentrations of 5 to 20% for cryo T and 0.25 to 1% for cryo P. These two products were tested in pairwise comparisons (Fig. 1).

Two packaging formats were prepared: (i) vials: 850 μl of the mix was dispensed into an 8 ml amber glass (VFA, Nanterre, France) vial and freeze-dried. The lyophilized mixture was reconstituted by adding 680 μl of sterile distilled water. The reconstituted solution (20 μl per well) was added to the test plate, together with 5 μl of purified DNA per well, giving a total volume of 25 μl per well; (ii) strips: 20 μl of the mixture was added to each well of strips suitable for use in a real-time thermal cycler and freeze-dried. The lyophilized mixture was reconstituted by adding 20 μl of sterile distilled water and 5 μl of purified DNA per well.

### Range of external *M*. *ulcerans* DNA standards

The range of external standards used in this study was generated from a mouse tail spiked with a known quantity of *M*. *ulcerans* (strain 1G897), extracted with the same extraction and purification protocol as was used for the tissue samples and serially diluted over 5 or 7 orders of magnitude, to concentrations of up to 1x10^6^ U/ml.

### Validation and characterization of dry qPCR mixes

The efficiency (i.e. yield), analytical sensitivities and accuracy of the various dry mixes were compared with those of fresh IS2404 qPCR mix, in quantitative assays on a range of external *M*. *ulcerans* DNA standards, serially diluted from 1x10^6^ to 1x10^**0**^ U/ml. Each mix was tested in triplicate, as previously described [14,15]. Dry mixes were considered unsuitable for diagnostic purposes if the threshold cycle (C_t_) was 0.15 log (0.5 C_t_) below the results obtained with the fresh mix, as described by the *Association Française de Normalisation* (NF T90-471).

### Evaluation of stability at high temperature

The stability of the various dry qPCR mixes at high temperature was evaluated, by exposing these mixes to a temperature of 50°C for two weeks. The detection efficiencies obtained were compared with that for the fresh mix, for the standard range of bacterial concentrations, from 1x10^6^ to 1x10^2^ U/ml.

### Diagnosis specimens

The specimens were collected by the local medical staff at the Pobè CDTUB. FNAs were collected from non-ulcerative lesions, swabs were taken from the undermined edges of the lesions and tissue specimens were obtained from surgically excised tissues, as described elsewhere [4,5,9,10,14,16,17].

### Clinical validation

The diagnosis sensitivities of the fresh mix and dry mix B were compared, on human samples, by reanalyzing 19 purified specimens (S2 Table) of various types from a retrospective study after their storage at −80°C. The specimens used were selected on the basis of their low bacterial content, as previously determined at Angers University Hospital (10 U/ml to 1000 U/ml, corresponding to ≈ 37.5 C_t_ and ≈ 30 C_t_, respectively). Diagnosis sensitivity was evaluated by comparing the results obtained with dry mix B and the reference method for these specimens.

During this study (January 2013—December 2013), analyses were carried out on 93 specimens suspected to contain *M*. *ulcerans* DNA and sent from the Pobè CDTUB (swabs, *n* = 48; tissue specimens, *n* = 27; FNAs, *n* = 18; S3 Table) for diagnosis. The specificity of dry mix B was assessed by comparing the results obtained with this mix with those obtained for the reference method, at the molecular biology laboratory of Angers University Hospital, which has been responsible for carrying out PCR diagnosis for Pobè since 2006.

### Internal quality control

Several controls were included, to exclude the possibility of contamination during the preparatory steps: (i) Extraction and purification controls were performed, with sterile water rather than a specimen for the negative control, and with an extract of the tail of a mouse inoculated with *M*. *ulcerans* containing 10^3^ bacteria for the positive control; (ii) As a negative qPCR mix control, 5 μl of elution buffer was added to the mixture in place of the template DNA; (iii) A range of external standard *M*. *ulcerans* DNA (strain 1G897) concentrations was used in each experiment, for quantification and to control for PCR efficiency.

Inhibition control reactions were performed to rule out the presence of inhibitors in the purified DNA.

A DNA extract from the standard range (2 μl of a 1x10^5^ U/ml DNA extract) was added to the mixtures, replacing 2 μl water. Inhibitory effects were excluded if the C_t_ was less than 0.15 log (0.5 C_t_) away from the expected results.

## Results

### Selection of the optimal cryoprotectant concentrations for the dry mix

**Fig 2:**
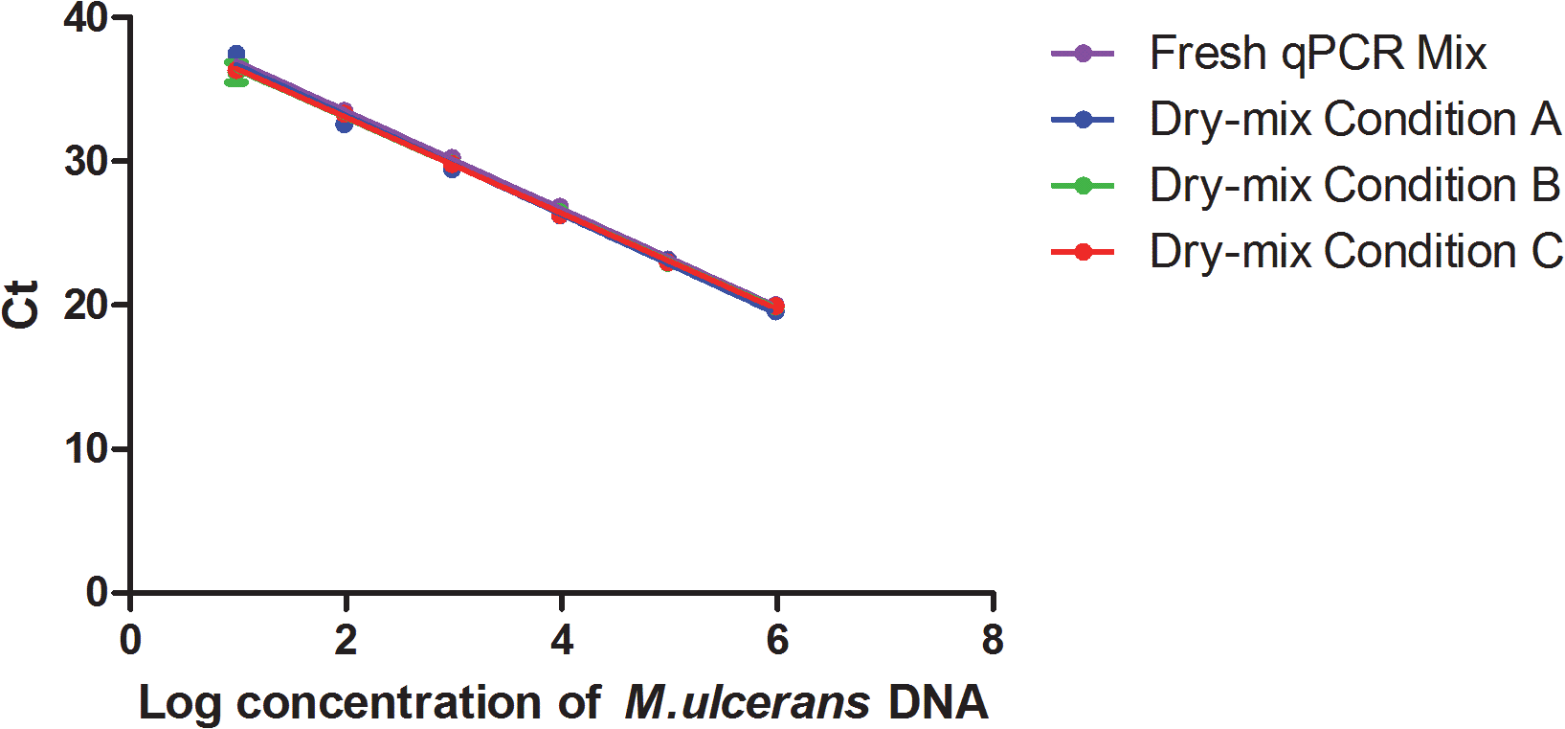
Comparison between 3 dry-reagent conditions and the standard reference method, based on an external standard curve. Serial 10-fold dilution from 1x10^6^ U/ml to 1x10^0^U/ml of *M*. *ulcerans* DNA. The serial curve was generated by a linear regression of the threshold cycle (C_t_) against the logarithm of *M*. *ulcerans* DNA concentration. Each 10-fold dilution was performed in triplicate. One standard deviation to either side of the mean is shown. Standard QPCR Mix refers to the reference method with fresh mix (*y* = −3.4107*x* + 40.357); dry-mix condition A refers to the dry mix containing 5% cryo T and 1% cryo P (*y* = −3.2333*x* + 39.087); dry-mix condition B refers to the dry mix containing 10% cryo T and 1% cryo P (*y* = −3.3727*x* + 39.942); dry-mix condition C refers to the dry mix containing 7.5% cryo T and 0.5% cryo P (*y* = −3.357*x* + 39.851). For each assay, the coefficient of correlation was greater than 0.99.

The minimum and maximum concentrations of the cryoprotectants compatible with the chosen qPCR technique were first determined. We used concentrations of 5 to 20% for cryo T and 0.25 to 1% for cryo P. These two products were tested in pairwise comparisons, to determine the optimal concentrations, using the external standard range obtained from mouse tails spiked with *M*. *ulcerans*. After preliminary evaluations, based on the efficiency of the dry mixes after storage at room temperature for 15 days, the conditions with suboptimal concentrations of cryoprotectant were discarded. Three conditions were retained for further investigations (Fig. 1). They were evaluated on the basis of four criteria: a comparison of the efficiencies of the dry mixes with that of the fresh mix, analytical sensitivity, accuracy and the visual appearance of the dry mixes. In qPCR assays, the results obtained with the fresh mix were significantly correlated with those obtained with the three dry mixes (Fig. 2). The cryoprotectants therefore had no inhibitory effect. Dry mixes A, B and C had efficiencies of 104%, 98% and 98%, respectively. No difference in accuracy was observed between the three dry mixes and the fresh mix (Δ*C*_*t*_<0.5).

The detection thresholds (mean C_t_) of the dry mixes A, B and C for the 1x10^1^ concentration of the DNA standard range were 37.47 (5 U/ml), 36.19 (12 U/ml) and 36.35 (11 U/ml), respectively, whereas that for the fresh mix was 36.95 (8 U/ml). No copy was detectable in any mix for the 1x10^0^ concentration. The analytical sensitivity was thus 10 U/ml and concentrations below 10 U/ml were considered detectable but inconsistent for quantification. The correlation coefficients were greater than 0.99 and the visual aspect of the dry products were conforming.

**Fig 3:**
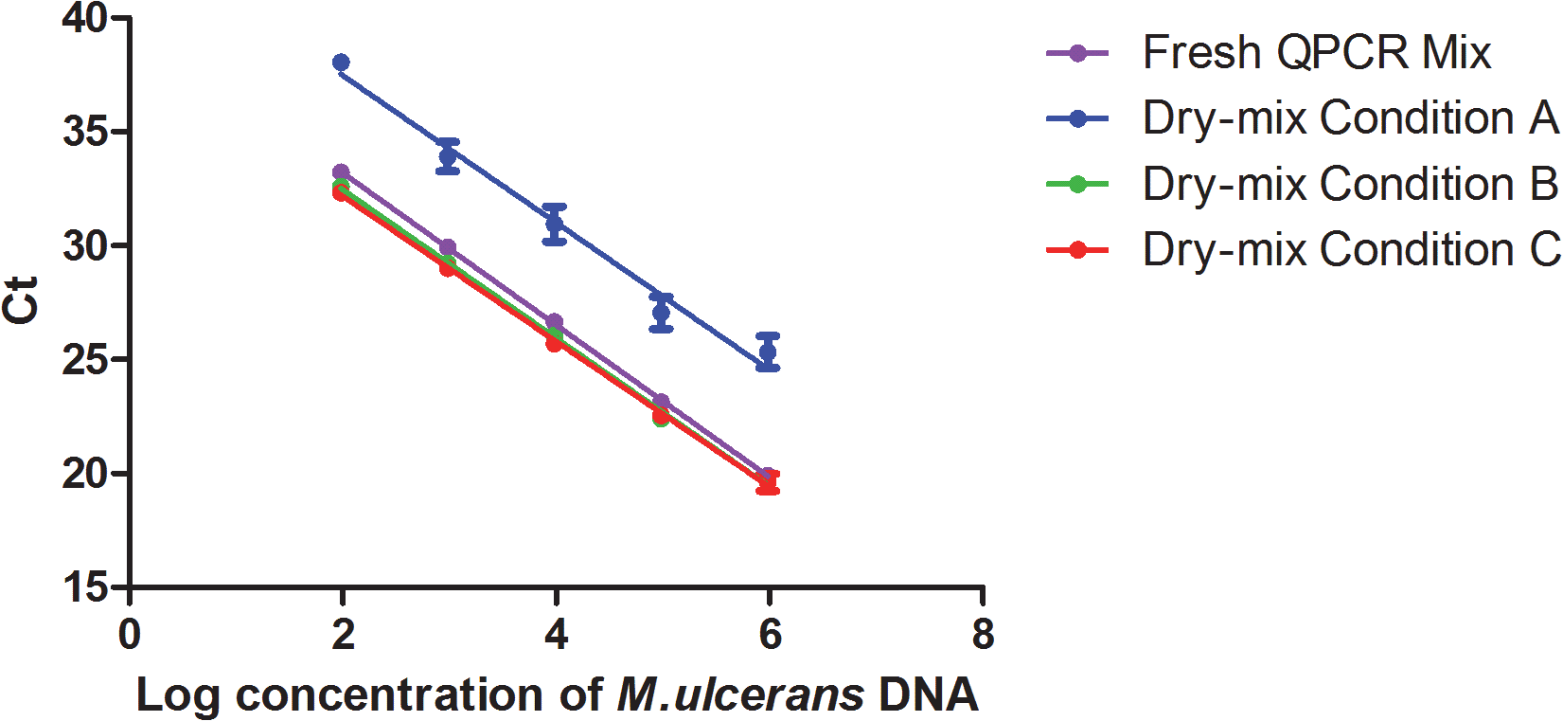
Comparison of the 3dry-reagents conditions after exposure to a temperature of 50°C for 15 days and the standard reference method, based on an external standard curve. Serial 10-fold dilutions, from 1x10^6^ U/ml to 1x10^2^ U/ml, of *M*. *ulcerans* DNA. The serial curve was generated by linear regression of the threshold cycle (C_t_) against the logarithm of *M*. *ulcerans* DNA concentration. Each 10-fold dilution was performed in triplicate. One standard deviation on either side of the mean is shown. “Standard QPCR Mix” corresponds to the reference method with fresh mix (*y* = −3.34*x* + 39.88); dry mix A is the dry mix containing 5% cryo T and 1% cryo P (*y* = −3.23*x* + 43.94); dry mix B is the dry mix containing 10% cryo T and 1% cryo P (*y* = −3.26*x* + 38.98); dry mix C is the dry mix containing 7.5% cryo T and 0.5% cryo P (*y* = −3.19*x* + 38.55).

Resistance to heat was therefore evaluated. The dry mixes were incubated at 50°C for two weeks, and their visual appearance was then assessed. Dry mix A looked shiny and retracted, whereas dry mix B still appeared to be dry, like the original freeze-dried product, and dry mix C appeared to have shrunk slightly in comparison to dry mix B. Dry mixes A, B and C had efficiencies of 104%, 103% and 106%, respectively, and correlation coefficients of 0.98, 0.99 and 0.99, which is acceptable. The qPCR results showed a lower accuracy for dry mix A after exposure to 50°C for 15 days than for the fresh mix, with a Δ*C*_*t*_ (dry mix A – fresh mix) between 3.93 and 5.42 (Fig. 3). After exposure to the same conditions, dry mixes B and C behaved similarly to the fresh mix (Δ*C*_*t*_<0.5). These results, indicating that dry mix A was not stable at high temperatures, resulted in the exclusion of this dry mix from subsequent experiments. Dry mixes B and C both gave good results after incubation at 50°C, but the slightly shrunken appearance of dry mix C reflected an inappropriate concentration of cryoprotectants. We therefore decided to use only dry mix B in subsequent studies.

### Validation of the analytical sensitivity of the selected dry mix against that of the fresh mix, for 19 selected weakly positive extracts

**Fig 4:**
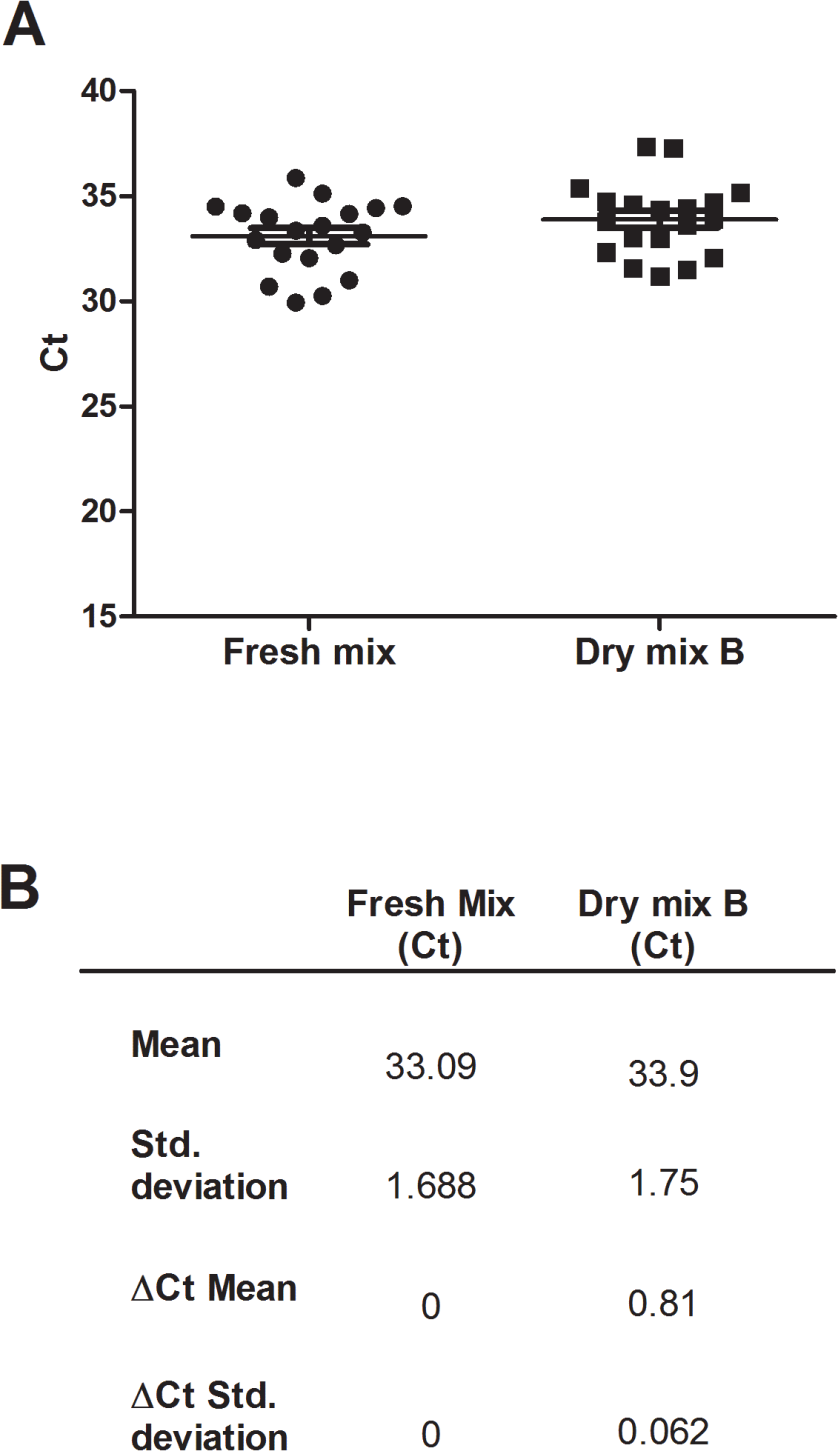
CT values for the 19 weakly positive samples, for 2 conditions. (A) C_t_ results for each sample obtained by the fresh mix and the dry mix B, Horizontal bars indicate mean values ± SD. (B) Mean values ± SD and average ΔC_t_ ± SD between fresh mix and dry mix B.

Nineteen purified DNA samples (S2 Table) with low concentrations were selected from a retrospective study for evaluation of the sensitivity of dry mix B. No differences in analytical sensitivity were found between the results obtained with the reference method and those obtained with the dry mixes, with 100% detection rates obtained with both methods. The ΔC_t_ value of the mean recorded for the quantification with the dry mix was more than 0.5 C_t_ (ΔC_t_ mean = 0.81) away from value for the fresh mix (Fig. 4), due to the imprecision introduced by the low copy number. No inhibitors were detected in the purified DNA during this experiment. The sensitivity of the dry mix was therefore similar to that of the fresh mix for use on clinical samples, even for the detection of small amounts of DNA.

### Clinical evaluation, comparing the selected dry mix with the fresh mix, for 93 patients with infection suspected on clinical grounds

**Fig 5:**
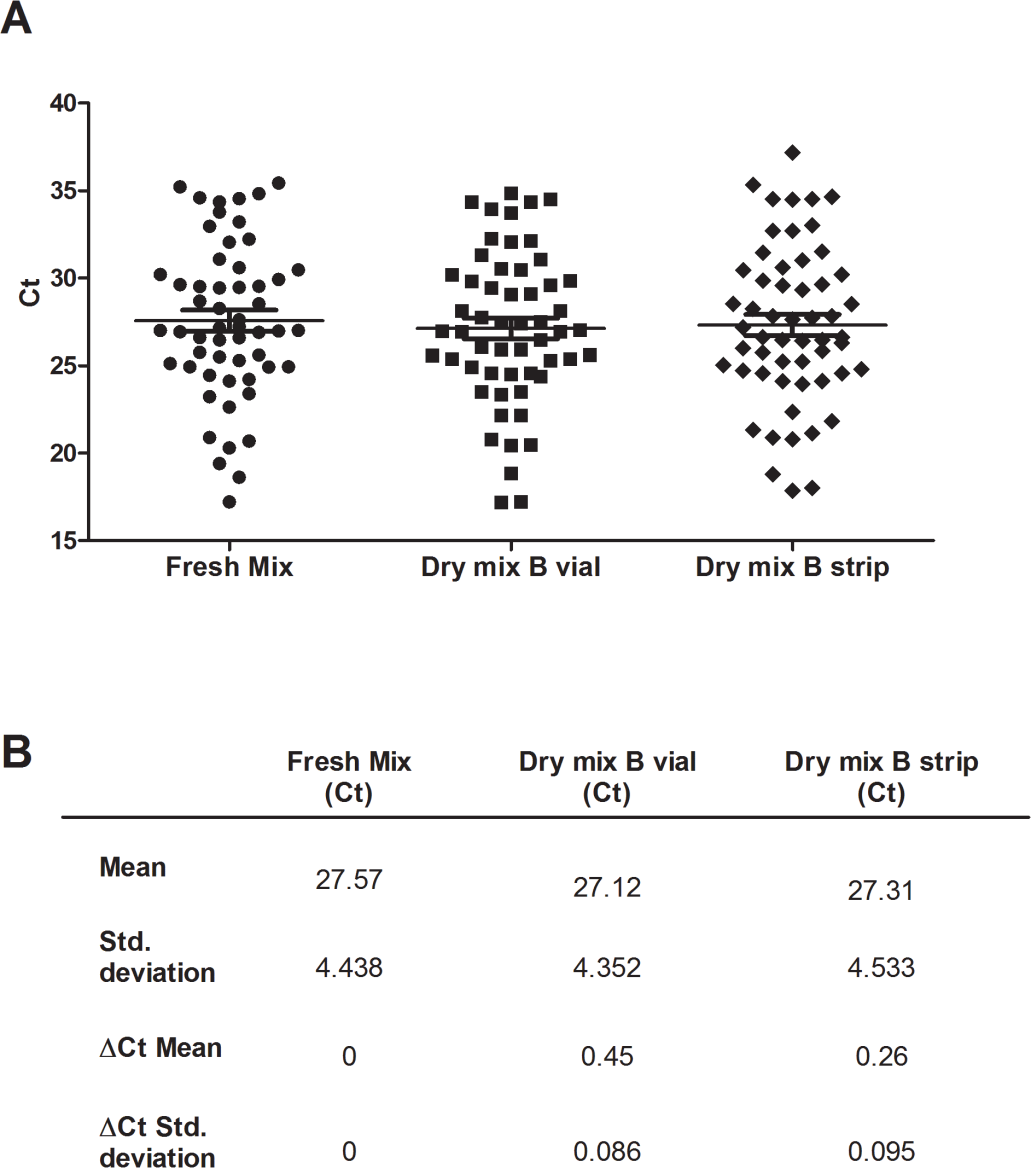
CT values for the 55 positive samples, for 3 mix conditions. (A) C_t_ results for each sample obtained by the fresh mix and the dry mix B in vial or in strip format, Horizontal bars indicate mean values ± SD. (B) Mean values ± SD and average ΔC_t_ ± SD between fresh mix and dry mix B in vial or in strip format.

For validation of the dry mix in field conditions, the selected dry mix was evaluated on 48 swabs, 27 tissue samples and 18 FNAs from 93 patients with suspected Buruli ulcer. Two different formats of the dry mix were also evaluated, for greater flexibility: (i) vials containing a volume sufficient for 32 reactions, for diagnostic tests on normal to large series of samples and (ii) eight-well strips, to decrease costs and facilitate the analysis of small series of samples. The results obtained with dry mix B, in the vial and strip formats, were compared with those obtained with the fresh mix. Identical results were obtained for all three types of test (S3 Table): 55 specimens were positive for *M*. *ulcerans* DNA and 38 were negative. The ΔC_t_ values recorded for the quantification with the dry mix were less than 0.5 C_t_ away from the values recorded with the fresh mix (Fig. 5). No inhibitors were detected in the purified DNA used. All cases testing positive were treated for Buruli ulcer. All subjects testing negative for Buruli ulcer were treated in accordance with the alternative diagnosis reached by the clinician. The diagnosis specificity of the two methods (dry mix and fresh mix) was 100%.

## Discussion

The WHO recommends IS*2404* qPCR amplification for the confirmation of Buruli ulcer diagnosis, because this technique is both the fastest and the most sensitive [9,10]. Many African countries have recently obtained qPCR equipment, for avian influenza surveillance. This equipment could also be used for the diagnosis of other diseases, including neglected diseases. However, the handling of sensitive reagents can be critical in tropical countries. This problem is jeopardizing the development of efficient diagnostic tools.

In this context, we developed a dry qPCR mix, overcoming local problems relating to transport, storage, cost and freezing/thawing issues, which can substantially lower the sensitivity of qPCR. Our dry-qPCR mix presents several major advantages over fresh qPCR mix. Its resistance to high temperatures should ensure its stability during handling in tropical conditions.

Furthermore, the number of new Buruli ulcer cases per week in African countries in which this disease is endemic is generally between 0.7 (Congo: 38 new cases in 2012) and 26.7 (Ivory Coast: 1386 new cases in 2012) [10]. Molecular diagnosis is therefore often delayed while additional samples are collected, so that molecular diagnostic tests can be carried out in batches, to decrease costs and the time spent on tests. The ready-to-use format simplifies handling by laboratory technicians, and the possibility of using either vials or microtube strips maximizes flexibility, enabling the laboratory to optimize time/sample and reagent/sample ratios. This simplification of handling decreases the time required for manipulations and minimizes the risk of contamination.

The dry mix qPCR approach could be adapted for other sets of primers and probes, such as the ketoreductase-B (KR) domain of the *M*. *ulcerans* mycolactone polyketide synthase genes. This would facilitate the diagnosis of many other neglected tropical diseases, such as rabies, trachoma and dengue.

## Supporting Information

S1 Table Primers and probe used to detect *M*. *ulcerans* DNA sequences by *Taq*Man real-time PCR. (DOCX)

S2 Table Listing of the weakly positive control samples and Ct results for the dry mix vs. gold standard method. “FNA” = fine needle aspiration, “U” = ulcerative forms, “E” = edematous forms, “Q” = plaques, “N” = nodules and two or more letters indicate mixed forms.
(DOCX)

S3 Table Listing of the 93 diagnosis specimens and Ct results for the two dry mixes vs. the gold standard method. “FNA” = fine needle aspiration, “U” = ulcerative forms, “E” = edematous forms, “Q” = plaques, “N” = nodules, “OS” = osteomyelitis and two or more letters indicate mixed forms.
(DOCX)

S1 Dataset Raw data of the QPCR with the different tested mixes. (XLSX)

S1 Text Document explaining S1 Dataset. (TXT)

## References

[1] Vincent QB, Ardant MF, Adeye A, Goundote A, Saint-Andre JP, Cottin J, et al (2014) Clinical epidemiology of laboratory-confirmed Buruli ulcer in Benin: a cohort study. Lancet Glob Health 2: e422–430. 10.1016/S2214-109X(14)70223-2 25103396

[2] Asiedu K, Sherpbier R, Raviglione MC (2000) Buruli Ulcer *Mycobacterium ulcerans* infection W.H.O. Global Buruli Ulcer initiative. Report 2000 World Health Organisation Geneva Switzerland.

[3] Johnson PD, Stinear T, Small PL, Pluschke G, Merritt RW, Portaels F, et al (2005) Buruli ulcer (*M*. *ulcerans* infection): new insights, new hope for disease control. PLoS Med 2: e108 15839744 10.1371/journal.pmed.0020108 PMC1087202

[4] Chauty A, Ardant MF, Adeye A, Euverte H, Guedenon A, Johnson C, et al (2007) Promising clinical efficacy of streptomycin-rifampin combination for treatment of buruli ulcer (*Mycobacterium ulcerans* disease). Antimicrob Agents Chemother 51: 4029–4035. 17526760 10.1128/AAC.00175-07 PMC2151409

[5] Chauty A, Ardant MF, Marsollier L, Pluschke G, Landier J, Adeye A, et al (2011) Oral treatment for *Mycobacterium ulcerans* infection: results from a pilot study in Benin. Clin Infect Dis 52: 94–96. 10.1093/cid/ciq072 21148526

[6] Portaels F, Agular J, Fissette K, Fonteyne PA, De Beenhouwer H, de Rijk P, et al (1997) Direct detection and identification of Mycobacterium ulcerans in clinical specimens by PCR and oligonucleotide-specific capture plate hybridization. J Clin Microbiol 35: 1097–1100. 9114387 10.1128/jcm.35.5.1097-1100.1997 PMC232709

[7] Rondini S, Mensah-Quainoo E, Troll H, Bodmer T, Pluschke G (2003) Development and application of real-time PCR assay for quantification of Mycobacterium ulcerans DNA. J Clin Microbiol 41: 4231–4237. 12958250 10.1128/JCM.41.9.4231-4237.2003 PMC193839

[8] Marion E, Ganlonon L, Claco E, Blanchard S, Kempf M, Adeye A, et al (2014) Establishment of qPCR and culture laboratory facilities in a field hospital in Benin: one year results. Journal of clinical Microbiology 52: 4398–4400 10.1128/JCM.02131-14 25320228 PMC4313327

[9] Portaels F, WorldHealthOrganization (2014) Laboratory diagnosis of buruli ulcer; A manual for health care providers In: Organization WH, editor. Geneva: World Health Organization pp. 105.

[10] WorldHealthOrganization (2013) Guidance on sampling techniques for laboratory-confirmation of Mycobacterium ulcerans infection (Buruli ulcer disease) Geneva: WHO.

[11] Siegmund V, Adjei O, Nitschke J, Thompson W, Klutse E, Herbinger KH, et al (2007) Dry reagent-based polymerase chain reaction compared with other laboratory methods available for the diagnosis of Buruli ulcer disease. Clin Infect Dis 45: 68–75. 17554703 10.1086/518604

[12] Siegmund V, Adjei O, Racz P, Berberich C, Klutse E, van Vloten F, et al (2005) Dry-reagent-based PCR as a novel tool for laboratory confirmation of clinically diagnosed Mycobacterium ulcerans-associated disease in areas in the tropics where M. ulcerans is endemic. J Clin Microbiol 43: 271–276. 15634982 10.1128/JCM.43.1.271-276.2005 PMC540149

[13] Rondini S, Mensah-Quainoo E, Junghanss T, Pluschke G (2006) What does detection of Mycobacterium ulcerans DNA in the margin of an excised Buruli ulcer lesion tell us? J Clin Microbiol 44: 4273–4275. 16928966 10.1128/JCM.00970-06 PMC1698343

[14] Cassisa V, Chauty A, Marion E, Ardant MF, Eyangoh S, Cottin J, et al (2010) Use of fine-needle aspiration for diagnosis of *Mycobacterium ulcerans* infection. J Clin Microbiol 48: 2263–2264. 10.1128/JCM.00558-10 20375229 PMC2884471

[15] Marion E, Eyangoh S, Yeramian E, Doannio J, Landier J, Aubry J, et al (2010) Seasonal and regional dynamics of *M*. *ulcerans* transmission in environmental context: deciphering the role of water bugs as hosts and vectors. PLoS Negl Trop Dis 4: e731 10.1371/journal.pntd.0000731 20625552 PMC2897839

[16] WorldHealthOrganization, editor (2012) Treatment of Mycobacterium ulcerans (Buruli ulcer): Guidance for health workers. Geneva. 73 p.

[17] Etuaful S, Carbonnelle B, Grosset J, Lucas S, Horsfield C, Phillips R, et al (2005) Efficacy of the combination rifampin-streptomycin in preventing growth of *Mycobacterium ulcerans* in early lesions of Buruli ulcer in humans. Antimicrob Agents Chemother 49: 3182–3186. 16048922 10.1128/AAC.49.8.3182-3186.2005 PMC1196249

