# Supplementary material for: Development of a Dry-Reagent-Based qPCR to Facilitate the Diagnosis of Mycobacterium ulcerans Infection in Endemic Countries
Source: PLoS Negl Trop Dis. 2015 Apr 1;9(4):e0003606. doi: 10.1371/journal.pntd.0003606 (PMC4382021; doi:10.1371/journal.pntd.0003606)
Supplement: S1 Table — (DOCX) [file pntd.0003606.s001.docx]

**Table S1: Primers and probe used to detect *M. ulcerans* DNA sequences by *Taq*Man real-time PCR**

| **Primer/probe name** | **Sequence (5' to 3')** |
| --- | --- |
| *IS*2404 forward primer | ATTGGTGCCGATCGAGTTG |
| *IS*2404 reverse primer | TCGCTTTGGCGCGTAAA |
| *IS*2404 probe | FAM-CACCACGCAGCATTCTTGCCGT-TAMRA |
