# Supplementary material for: Development of a Dry-Reagent-Based qPCR to Facilitate the Diagnosis of Mycobacterium ulcerans Infection in Endemic Countries
Source: PLoS Negl Trop Dis. 2015 Apr 1;9(4):e0003606. doi: 10.1371/journal.pntd.0003606 (PMC4382021; doi:10.1371/journal.pntd.0003606)
Supplement: S2 Table — “FNA” = fine needle aspiration, “U” = ulcerative forms, “E” = edematous forms, “Q” = plaques, “N” = nodules and two or more letters indicate mixed forms. (DOCX) [file pntd.0003606.s002.docx]

**Table S2: Listing of the weakly positive control samples and Ct results for the dry mix vs. gold standard method**

“FNA” corresponds to fine needle aspiration, “U” corresponds to ulcerative forms, “E” corresponds to edematous forms, “Q” corresponds to plaques, “N” corresponds to nodules and two or more letters corresponds to mixed forms.

| **Low positive**  **control sample** | **Prelevement**  **type** | **Lesion**  **type** | **Standard Mix**  **(Ct)** | **Dry-mix**  **(Ct)** |
| --- | --- | --- | --- | --- |
| **1** | Tissue | UQ | 31 | 35.39 |
| **2** | FNA | EQ | 32.67 | 33.63 |
| **3** | Tissue | U | 32.26 | 32.34 |
| **4** | FNA | Q | 34.15 | 34.35 |
| **5** | Swab | EU | 34.52 | 37.34 |
| **6** | Swab | U | 33.99 | 35.15 |
| **7** | Swab | EU | 35.86 | 37.27 |
| **8** | Swab | UC | 32.06 | 32.99 |
| **9** | FNA | NU | 30.26 | 31.19 |
| **10** | Swab | U | 30.69 | 34.43 |
| **11** | Swab | NU | 34.51 | 34.59 |
| **12** | Swab | UQ | 33.36 | 34.73 |
| **13** | FNA | EQ | 33.58 | 33.02 |
| **14** | Swab | UQ | 34.44 | 34.72 |
| **15** | Tissue | U | 32.92 | 33.91 |
| **16** | FNA | UQ | 33.27 | 33.78 |
| **17** | FNA | EQ | 35.13 | 32.07 |
| **18** | Swab | U | 29.93 | 31.6 |
| **19** | Swab | UE | 34.18 | 31.51 |
