# Supplementary material for: Development of a Dry-Reagent-Based qPCR to Facilitate the Diagnosis of Mycobacterium ulcerans Infection in Endemic Countries
Source: PLoS Negl Trop Dis. 2015 Apr 1;9(4):e0003606. doi: 10.1371/journal.pntd.0003606 (PMC4382021; doi:10.1371/journal.pntd.0003606)
Supplement: S3 Table — “FNA” = fine needle aspiration, “U” = ulcerative forms, “E” = edematous forms, “Q” = plaques, “N” = nodules, “OS” = osteomyelitis and two or more letters indicate mixed forms. (DOCX) [file pntd.0003606.s003.docx]

**Table S3: Listing of the 93 diagnosis specimens and Ct results for the two dry mixes vs. the gold standard method**

“FNA” corresponds to fine needle aspiration, “U” corresponds to ulcerative forms, “E” corresponds to edematous forms, “Q” corresponds to plaques, “N” corresponds to nodules, “OS” corresponds to osteomyelitis and two or more letters corresponds to mixed forms.

| **Positive samples** | **Sampling method** | **Clinical form** | **Standard mix (Ct)** | **Dry mix B vial (Ct)** | **Dry mix B strips (Ct)** |
| --- | --- | --- | --- | --- | --- |
| **1** | swab | UEQ | 28.26 | 27.41 | 27.62 |
| **2** | FNA | EQ | 32.23 | 31.08 | 31.02 |
| **3** | swab | UEQ | 23.22 | 22.16 | 22.35 |
| **4** | FNA | Q | 25.61 | 23.51 | 24.12 |
| **5** | FNA | Q | 26.93 | 25.59 | 25.84 |
| **6** | tissue | UEQ | 20.89 | 20.8 | 20.8 |
| **7** | swab | UEQ | 27.63 | 27.03 | 26.4 |
| **8** | swab | Q | 20.69 | 20.46 | 20.89 |
| **9** | tissue | U | 17.21 | 17.21 | 17.85 |
| **10** | swab | UQ | 28.69 | 27.46 | 28.25 |
| **11** | swab | UQ | 30.47 | 30.2 | 30.6 |
| **12** | swab | UQ | 27.01 | 26.93 | 26.47 |
| **13** | swab | UQ | 25.12 | 24.58 | 24.79 |
| **14** | swab | U | 27.16 | 26.09 | 26.47 |
| **15** | swab | OS | 27.24 | 26.96 | 26.63 |
| **16** | tissue | OS | 30.6 | 29.46 | 30.21 |
| **17** | swab | UQ | 26.59 | 27.75 | 27.18 |
| **18** | tissue | U | 19.4 | 18.86 | 18.8 |
| **19** | swab | U | 31.08 | 31.34 | 31.45 |
| **20** | swab | UQ | 20.3 | 20.47 | 21.32 |
| **21** | tissue | UQ | 18.62 | 17.18 | 18.02 |
| **22** | swab | UQ | 33.21 | 32.09 | 32.69 |
| **23** | swab | UQ | 24.45 | 23.53 | 24.72 |
| **24** | FNA | E | 29.92 | 29.86 | 32.7 |
| **25** | swab | UEQ | 23.4 | 23.37 | 21.83 |
| **26** | FNA | EQ | 29.44 | 29.07 | 29.57 |
| **27** | tissue | U | 34.55 | 34.37 | 35.32 |
| **28** | tissue | U | 25.76 | 25.29 | 25.99 |
| **29** | swab | UQ | 34.36 | 34.51 | 37.17 |
| **30** | FNA | Q | 28.53 | 27.5 | 27.82 |
| **31** | FNA | UEQ | 25.3 | 24.57 | 24.56 |
| **32** | swab | UEQ | 24.12 | 25.4 | 25.03 |
| **33** | FNA | E | 30.21 | 29.61 | 29.63 |
| **34** | swab | U | 35.21 | 33.73 | 34.66 |
| **35** | swab | UQ | 24.21 | 24.38 | 24.1 |
| **36** | FNA | E | 34.83 | 33.97 | 34.53 |
| **37** | swab | EQ | 26.47 | 25.94 | 26.3 |
| **38** | swab | U | 25.49 | 25.93 | 25.74 |
| **39** | tissue | U | 26.91 | 26.93 | 27.72 |
| **40** | swab | U | 35.44 | 34.88 | 34.5 |
| **41** | FNA | E | 34.59 | 34.35 | 34.52 |
| **42** | swab | EUQ | 27.02 | 26.46 | 26.62 |
| **43** | swab | U | 32.97 | 32.13 | 31.5 |
| **44** | FNA | EQ | 29.53 | 28.12 | 28.51 |
| **45** | FNA | E | 33.77 | 32.27 | 33.01 |
| **46** | tissue | Q | 26.61 | 25.62 | 25.25 |
| **47** | swab | UQ | 24.92 | 24.53 | 25.23 |
| **48** | swab | U | 29.48 | 29.11 | 28.53 |
| **49** | FNA | E | 29.63 | 28.12 | 27.83 |
| **50** | tissue | U | 24.93 | 24.92 | 23.95 |
| **51** | swab | U | 24.94 | 25.41 | 24.55 |
| **52** | tissue | U | 22.63 | 22.18 | 21.13 |
| **53** | swab | U | 32.06 | 30.55 | 29.85 |
| **54** | tissue | U | 26.98 | 30.47 | 30.45 |
| **55** | swab | U | 29.52 | 29.82 | 29.33 |
|  |  |  |  |  |  |
| **Negative samples** | **Specimen type** | **Lesion type** | **Standard mix (Ct)** | **Dry-mix B vial (Ct)** | **Dry-mix B strips (Ct)** |
| **1** | swab | OS | Not detected | No detected | No detected |
| **2** | swab | UEQ | Not detected | No detected | No detected |
| **3** | tissue | U | Not detected | No detected | No detected |
| **4** | swab | U | Not detected | No detected | No detected |
| **5** | tissue | U | Not detected | No detected | No detected |
| **6** | swab | UC | Not detected | No detected | No detected |
| **7** | tissue | U | Not detected | No detected | No detected |
| **8** | swab | UQ | Not detected | No detected | No detected |
| **9** | swab | U | Not detected | No detected | No detected |
| **10** | tissue | U | Not detected | No detected | No detected |
| **11** | swab | U | Not detected | No detected | No detected |
| **12** | swab | U | Not detected | No detected | No detected |
| **13** | swab | UQ | Not detected | No detected | No detected |
| **14** | swab | UQ | Not detected | No detected | No detected |
| **15** | swab | U | Not detected | No detected | No detected |
| **16** | FNA | E | Not detected | No detected | No detected |
| **17** | tissue | OS | Not detected | No detected | No detected |
| **18** | tissue | U | Not detected | No detected | No detected |
| **19** | swab | U | Not detected | No detected | No detected |
| **20** | FNA | E | Not detected | No detected | No detected |
| **21** | tissue | OS | Not detected | No detected | No detected |
| **22** | tissue | OS | Not detected | No detected | No detected |
| **23** | tissue | OS | Not detected | No detected | No detected |
| **24** | FNA | UQ | Not detected | No detected | No detected |
| **25** | swab | U | Not detected | No detected | No detected |
| **26** | tissue | U | Not detected | No detected | No detected |
| **27** | swab | U | Not detected | No detected | No detected |
| **28** | swab | U | Not detected | No detected | No detected |
| **29** | FNA | N | Not detected | No detected | No detected |
| **30** | FNA | E | Not detected | No detected | No detected |
| **31** | swab | U | Not detected | No detected | No detected |
| **32** | tissue | OS | Not detected | No detected | No detected |
| **33** | tissue | OS | Not detected | No detected | No detected |
| **34** | swab | UQ | Not detected | No detected | No detected |
| **35** | tissue | U | Not detected | No detected | No detected |
| **36** | swab | OS | Not detected | No detected | No detected |
| **37** | tissue | OS | Not detected | No detected | No detected |
| **38** | swab | UQ | Not detected | No detected | No detected |
